# Overall survival of patients with KIT-mutant metastatic GIST in the era of multiple kinase inhibitor availability

**DOI:** 10.1007/s00432-024-05965-2

**Published:** 2024-11-09

**Authors:** Valerie Haller, Carina Reiff, Rainer Hamacher, Karina Kostbade, Moritz Kaths, Juergen Treckmann, Stefanie Bertram, Yasmin Zaun, Sebastian Bauer, Johanna Falkenhorst

**Affiliations:** 1 Department of Medical Oncology and Sarcoma Center, West German Cancer Center, University Hospital Essen, Essen, Germany; 2 German Cancer Consortium (DKTK), Partner Site University Hospital Essen, Essen, Germany; 3 Department of Visceral and Transplantation Surgery, University Hospital Essen, Essen, Germany; 4 Department of Pathology, University Hospital Essen, Essen, Germany

## Abstract

**Purpose:**

The prognosis of patients with metastatic GIST and imatinib-sensitive primary mutations has significantly improved. However, limited data are available to inform patients about outcomes across different lines of treatment. This retrospective analysis aims to evaluate patient outcomes at a large German GIST referral center over the past 15 years.

**Patients and methods:**

Overall survival (OS) and progression-free survival (PFS) were analyzed in patients with metastatic GIST, with diagnosis of metastases between 2008 and 2021, when at least three lines of treatment were available in Germany (*n* = 174).

**Results:**

The median overall survival far exceeds historical data for patients with primary exon 11 and exon 9 mutations (median OS in palliative treatment with imatinib: 7.1 years; median OS in second-line palliative treatment with sunitinib: 2.9 years; median OS in third-line palliative treatment with regorafenib: 1.9 years). Among those patients who received palliative imatinib treatment, no significant difference in median OS survival was observed between those who had received perioperative imatinib for localized disease and those who did not. Furthermore, the location of metastases significantly impacted survival, whereas the time between the initial diagnosis and the diagnosis of metastases had no significant effect on survival.

**Conclusion:**

In conclusion, this study provides a novel, real-world reference for survival outcomes in patients with metastatic GIST.

## Introduction

Gastrointestinal stromal tumors (GIST) are the most prevalent gastrointestinal sarcomas and represent one of the most frequent soft tissue sarcoma subtypes overall (de Pinieux et al. 2021). The majority of cases exhibit recurrent oncogenic mutations of the KIT and PDGFRA receptor tyrosine kinases (Corless et al. 2011), which are associated with a unique oncogenic addiction. Inhibitors of KIT and PDGFRA represent the only therapeutic approach for these patients and provide a highly effective mechanism-based treatment. To date, five treatments have been approved for GIST: imatinib, sunitinib, regorafenib and ripretinib for KIT-mutant GIST and avapritinib for patients with D842V-mutant GIST (Demetri et al. 2002, 2006, 2012b; Blay et al. 2020; Heinrich et al. 2020).

In patients for whom a medical treatment is being considered, genotyping is the standard of care prior to treatment initiation (Casali et al. 2022). For KIT-mutant GIST, the most common primary mutations are found in the juxtamembrane domain (encoded by exon 11) and in the extracellular (dimerization) domain (encoded by exon 9). Exon 11 mutations are invariably sensitive to all approved KIT inhibitors, whereas exon 9 mutations show a slightly decreased sensitivity to imatinib and ripretinib (Bauer et al. 2022; MetaGIST 2010). For imatinib, dose-escalation up to 800 mg daily has been associated with improved objective response rates and progression-free survival in patients with metastatic disease (Casali et al. 2022). For patients with imatinib-sensitive KIT mutations, median progression-free survival to first-line therapy is approximately 18 to 24 months, depending on the genotype (MetaGIST 2010). While imatinib can achieve long-term disease control in about 10% of patients, most patients with metastatic disease will eventually progress (Blanke et al. 2008; Patel 2013). The main mechanisms of resistance identified in tumor lesions progressing on imatinib are secondary mutations within the ATP-binding pocket or the activation loop of KIT (encoded by exons 13/14 and 17/18, respectively) (Heinrich et al. 2006, 2024). Recently, more complex compound mutations have been described in patients progressing on ripretinib, characterized by mutations within the ATP-binding pocket (AP) and activation loop (AL) domains on the same allele (in cis) (Mühlenberg et al. 2024).

For patient counseling, survival data are mostly based on international registration trials, that do not fully represent the real-life patient cohorts or include novel local and systemic treatment options. Many patients in the early imatinib-trials had started treatment with a larger tumor burden than is typically seen today, particularly in those with metachronous metastases. Patients who progressed on imatinib in the early 2000s had few alternative treatment options, and even in the past 10 years, many patients taking part in international registration trials had limited access to advanced treatment modalities.

The following study analyzes a large cohort (*n* = 174) regarding the outcomes of the approved first three treatment lines in GIST (imatinib, sunitinib, regorafenib). The data were acquired from the West German Cancer Center, representing a healthcare system with typically universal access to approved treatments, regardless of socio-economic status. The cohort reflects a time period in which at least three lines of therapy were available, providing a real-world reference for patient counseling in Germany.

## Methods

**Fig. 1:**
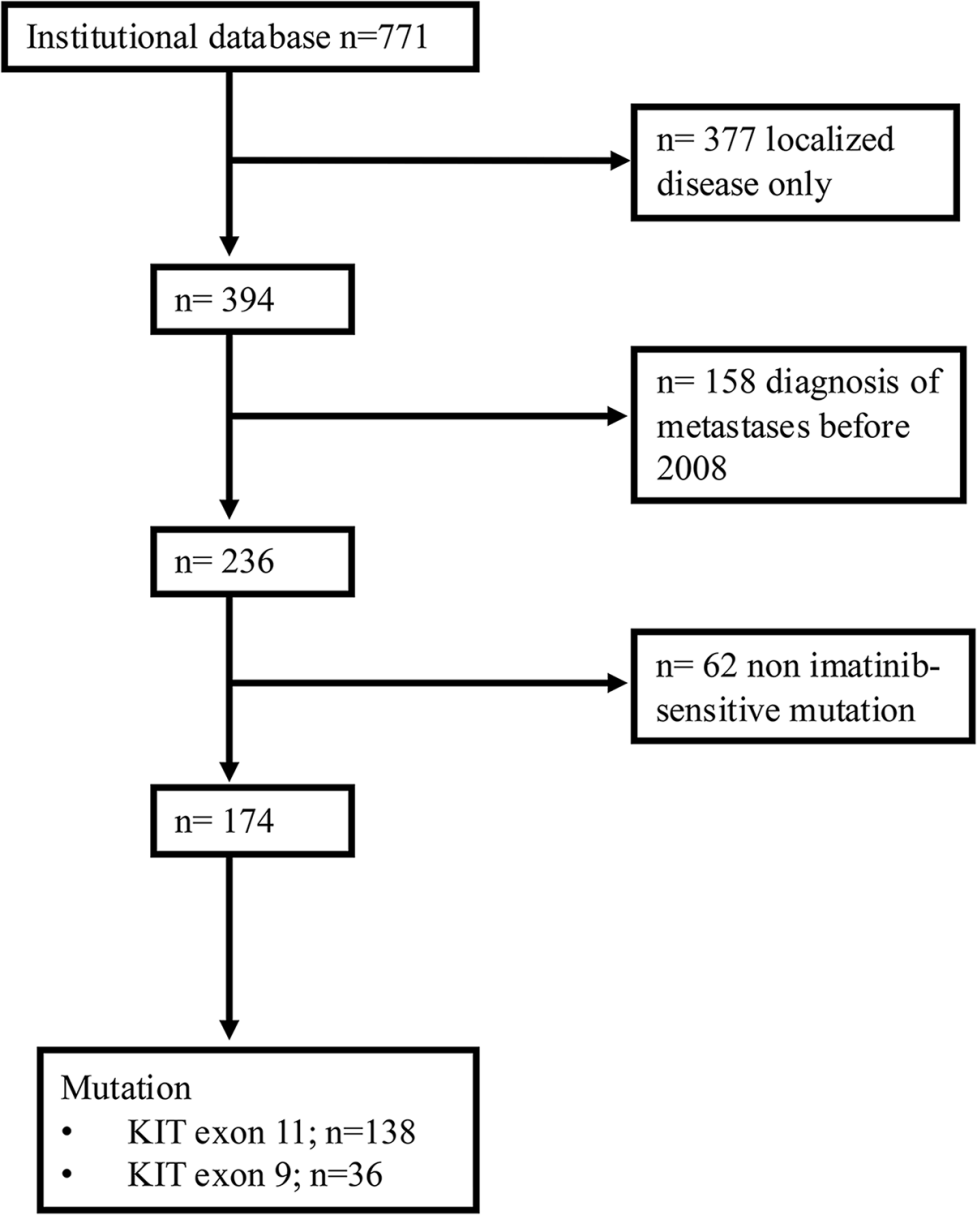
CONSORT diagram. The institutional database comprised a total of *n* = 771 patients. Among these, 377 patients with localized disease were excluded from the analysis. Additionally, 158 patients whose diagnosis of metastatic disease predated 2008 were also excluded. Furthermore, 62 patients lacked an imatinib-sensitive mutation, resulting in a final cohort size of *n* = 174 patients. Among these, *n* = 138 harbored primary KIT exon 11 mutations, while *n* = 36 exhibited primary KIT exon 9 mutations

The institutional GIST database (*n* = 771) of the Sarcoma Center at the Essen University Hospital was queried for patients with metastatic disease (*n* = 394) diagnosed between 2008 and 2021 (*n* = 236). Patients with primary *KIT* exon 17 mutations, SDH-deficient GIST, NF-1 associated GIST, as well as those with a primary PDGFRA D842V mutation, were excluded, resulting in a total of 174 patients with primary exon 11 (*n* = 138) or primary exon 9 mutation (*n* = 36; Fig. 1).

The median follow-up time since the diagnosis of metastases in this dataset was 4.7 years (0.1–14.5 years, Table 1). Patient data were obtained from the electronic hospital information system (HIS) of the Essen University Hospital. The project was approved by the institutional Ethics Committee of the University of Duisburg-Essen, Germany (15-6339-BO), and was conducted in accordance with the Declaration of Helsinki. All analyses were performed using the SPSS 29.0.1.0 software.

Overall Survival (OS) was defined as the length of time in years, from the first day of kinase-inhibitor treatment until death from any cause. Patients still alive were censored at the time of last available follow-up. Progression Free Survival (PFS) was calculated in years as the time from the initiation of imatinib treatment to the clinical progression of disease, typically triggering the next line of treatment. OS and PFS estimates, along with standard errors, were determined and mapped using the Kaplan-Meier method. Statistical comparisons were performed using the log-rank test, with a *p-*value of less than 0,05 considered statistically significant. Metachronous metastases were defined as metastases occurring more than six months after the diagnosis of the primary tumor.

## Results

**Table 1:** Description of demographics

**Gender**	n = 174
• Male	n = 103 (59%)
• Female	n = 71 (41%)
**Follow-up**	n = 174
• Alive	n = 97 (56%)
• Dead	n = 77 (44%)
o Tumor-related death	n = 71 (92.2%)
o No tumor-related death	n = 6 (7.8%)
▪ Cardio-vascular events	n = 4 (66.8%)
▪ Unknown, but regressive tumor findings under continued therapy	n = 1 (16.6%)
▪ Local complication under non-GIST-associated secondary tumor (cervical squamous cell carcinoma)	n = 1 (16.6%)
**Age at time of diagnosis** (years) (n = 174)	Median 57 (27–80)
**Age at time of diagnosis of metastases** (years) (n = 174)	Median 59 (27–82)
**Year of diagnosis of metastases** (n = 174)	2008–2021
**Follow up since diagnosis (years)** (n = 174)	Median 6.6 years
• Minimum	1 year
• Maximum	25.5 years
**Follow up since diagnosis of metastases** (years) (n = 174)	Median 4.7 years
• Minimum	0.1 year
• Maximum	14.5 years
**Follow up since first appointment in patients with metastatic disease** (years) (n = 174)	Median 2.54 years
• Minimum	0 months
• Maximum	14.2 years
**Primary mutations**	n = 174
• KIT exon 11	n = 138 (79%)
• KIT exon 9	n = 36 (21%)
**Location of primary**	n = 174
• Gastric	n = 60 (34.5%)
• Small intestine	n = 93 (53.5%)
• Colon	n = 9 (5%)
• Other (not specified)	n = 12 (7%)
**Location of metastases**	n = 174
• Peritoneum	n = 67 (38.5%)
• Liver	n = 64 (36.8%)
• Liver and Peritoneum	n = 40 (23%)
• Other (pancreas, small intestine)	n = 3 (1.7%)
**Time to diagnosis of metastases**	n = 174
• Metachronous	n = 85 (48.9%)
• Synchronous	n = 89 (51.1%)
**Time to diagnosis of metastases** (in months)	Median 5 (0-210 months)
**Resection of primary**	n = 174
• Yes	n = 146 (84%)
• No	n = 28 (16%)
**Metastasectomy**	n = 174
• Yes	n = 96 (55.2%)
• Location of metastases	
o Liver	n = 35 (36.5%)
o Peritoneum	n = 38 (39.6%)
o Liver and peritoneum	n = 22 (22.9%)
o Missing	n = 1 (1%)
• No/TKI-treatment only	n = 78 (44.8%)
**Perioperative imatinib therapy for initial localized disease**	n = 75
o Excluded patients (resulting in n = 75 patients analyzed for perioperative imatinib therapy for initial localized disease)	
▪ Patients who received adjuvant imatinib but discontinued treatment after less than 5 months due to toxicities	n = 7
▪ Patients who received additive therapy after an initial tumor rupture	n = 3
• Perioperative imatinib treatment	n = 48 (64%)
o Relapse Risk in localized disease	
▪ 0–40%	n = 4 (8.3%)
▪ 40–60%	n = 5 (10.4%)
▪ 60–80%	n = 8 (16.7%)
▪ 80–90%	n = 7 (14.6%)
▪ 90–100%	n = 13 (27.1%)
▪ Missing	n = 11 (22.9%)
• No perioperative imatinib treatment	n = 27 (36%)
o Relapse Risk in localized disease	
▪ 0–40%	n = 7 (25.9%)
▪ 40–60%	n = 3 (11.1%)
▪ 60–80%	n = 1 (3.7%)
▪ 80–90%	n = 2 (7.4%)
▪ 90–100%	n = 5 (18.5%)
▪ Missing	n = 9 (33.4%)
• Reason for no perioperative imatinib therapy	
o Treatment not offered	n = 25 (92.6%)
o Patient refused treatment	n = 1 (3.7%)
o Unknown	n = 1 (3.7%)
**Time between initial diagnosis and first appointment** (in months) (n = 172)	Median 27 (0-290 months)
**Place for primary treatment**	n = 174
• Local hospital	n = 153 (87.9%)
• Sarcoma center	n = 12 (6.9%)
• Practicing oncologist	n = 9 (5.2%)
**Place of treatment for metastatic disease**	n = 174
• Local hospital	n = 102 (58.6%)
• Sarcoma center	n = 64 (36.8%)
• Practicing oncologist	n = 8 (4.6%)
**State of disease at first appointment**	n = 174
• First diagnosis of metastatic disease	n = 32 (18.4%)
• External diagnosis of metastatic disease	n = 142 (81.6%)
**Palliative treatment lines** (n = 174)	Median 4 (1–8)
**Number of treatment lines in patients who died of their disease**	n = 71
• Range	1–8
• Median	4
o 1 treatment line	n = 2 (2.8%) (cum. 100%)
o 2 treatment lines	n = 6 (8.5%) (cum. 97.2%)
o 3 treatment lines	n = 7 (9.8%) (cum. 88.7%)
o 4 treatment lines	n = 22 (31%) (cum. 78.9%)
o 5 treatment lines	n = 19 (26.8%) (cum. 47.9%)
o >=6 treatment lines	n = 15 (21.1%) (cum. 21.1%)
**Number of treatment lines in patients with no tumor-related death**	n = 6
• Range	1–5
• Median	2
o 1 treatment line	n = 2 (33.3%)
o 2 treatment lines	n = 2 (33.3%)
o 4 treatment lines	n = 1 (16.7%)
o 5 treatment lines	n = 1 (16.7%)
**Medication as part of clinical trial at any point during treatment**	n = 174
• Yes	n = 50 (28.7%)
o Patients with tumor-related death	n = 33 (66%)
• No	n = 124 (71.3%)

### Patient and disease characteristics

In this retrospective cohort study, data from 174 patients diagnosed with metastatic GIST were analyzed. Of these, 59% (*n* = 103) were male and 41% (*n* = 71) were female. The median age at diagnosis was 57 years (range 27–80 years), and the median age at the diagnosis of metastatic disease was 59 years (range 27–82 years). The median follow-up time since the diagnosis of metastases was 4.7 years (range 0.1–14.5 years).

Small intestinal GIST constituted the majority (53.5%, *n* = 93), followed by gastric GIST (34.5%, *n* = 60), and other less common primary sites (12%, *n* = 21). Metastatic disease occurred within the peritoneum (38.5%, *n* = 67) or liver (36.8%, *n* = 64), while 23% (*n* = 40) exhibited both hepatic and peritoneal metastases. An additional 1.7% (*n* = 3) presented with metastases in other, less common sites. At the time of initial diagnosis, metastases were present in 51.1% (*n* = 89) of patients, while 48.9% (*n* = 85) developed metachronous metastases. The median interval between the diagnosis of the primary disease and the identification of metastases was five months (range 0-210 months).

The median time from the first diagnosis to the first visit at our site was 27 months (0-290 months). Primary treatment was mostly conducted in local hospitals (87.9%, *n* = 153), with 6.9% (*n* = 12) receiving treatment in sarcoma centers. Notably, at time of diagnosis of metastatic disease, the involvement of sarcoma centers increased to 36.8% (*n* = 64) (Table 1).

### Treatment and follow-up

Resection of the primary tumor was performed in 84% (*n* = 146) of patients, and 55.2% (*n* = 96) of patients underwent a metastasectomy at some point during their treatment. Patients received a median of four lines of therapy with tyrosine kinase inhibitors. In our cohort, 77 (44%) patients died during follow-up, with 71 (92.2%) of these deaths attributed to tumor-related causes. Notably, 7.8% (*n* = 6) of deaths were due to causes unrelated to GIST (mostly cardio-vascular events such as thromboembolic incidents or heart failure/myocardial infarction). These patients died during first line (*n* = 2), during second line (*n* = 2), during 4th line (*n* = 1) and one was a patient in 5th line treatment while still responding to therapy. Among those who died from their disease, 21.1% received up to three treatment regimens, 31% received four, 26.8% received five, and 21.1% received six or more. 88.7% of patients who had died from their disease had received at least three lines of treatment. 28.7% (*n* = 50) of all patients in the cohort participated in a clinical trial at some point during their treatment. Of those patients who died from their disease, 47% (*n* = 33) had participated in a clinical trial (Table 1).

### Overall survival by treatment line

**Fig. 2:**
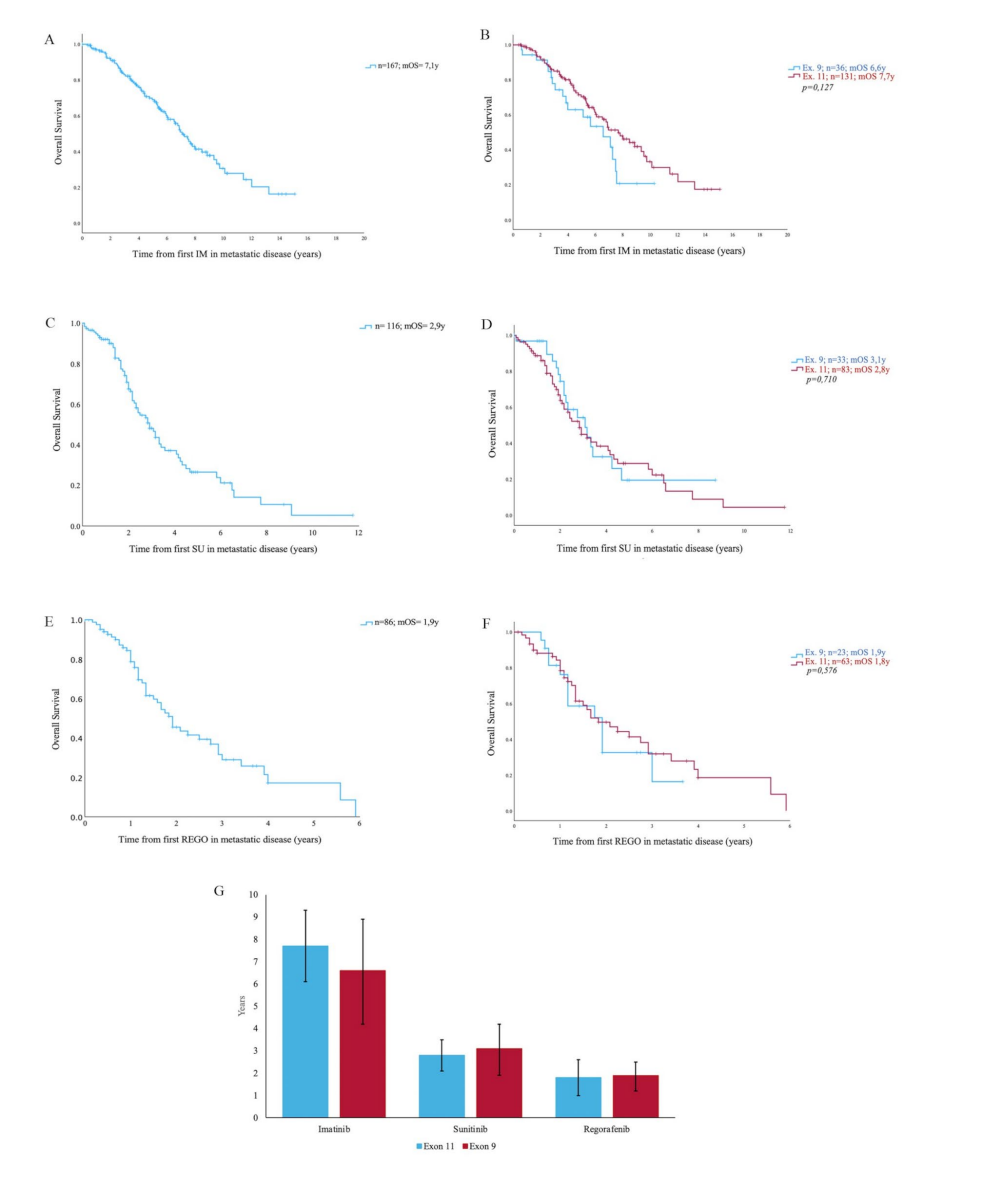
Kaplan-Meier estimation for overall survival **A** and **B**): Calculated from the first treatment with imatinib (IM) for metastatic GIST until last follow-up (FU) or death (2**A**) and comparison of two cohorts: Primary KIT exon 9 or KIT exon 11 mutation (2**B**). **C** and **D**): Kaplan-Meier estimation from the first treatment with sunitinib (SU) (2**C**) and comparison of two cohorts (2**D**). **E** and **F**): Kaplan-Meier estimation from the first treatment with regorafenib (REGO) (2E) also divided into two cohorts (2**F**). **G**): Median overall survival in years based on palliative treatment lines in primary exon 11 and exon 9 mutant GIST, with confidence intervals (2**G**)

The median overall survival (mOS) for patients receiving imatinib for metastatic disease was 7.1 years (0-15.1 years, 95% CI [6.2-8.0]; Fig. 2A, B). When calculated from start of second-line treatment (mainly sunitinib) mOS was 2.9 years (0-11.75 years, 95% CI [2.27–3.56]; Fig. 2C, D). For patients starting (mostly as third line) therapy with regorafenib, the median overall survival was 1.9 years (range 0-5.9 years, 95% CI [1.49–2.35]; Fig. 2E, F). For all treatment lines, no differences were observed between exon 11 and exon 9 mutant GIST (imatinib: 7.7 years (0-15.1 years; 95% CI [6.1–9.3]) vs. 6.6 years (0-10.3years; 95% CI [4.2–8.9]); *p* = 0.127; 2nd line: 2.8 years (0-11.75 years; 95% CI [2.1–3.5]) vs. 3.1years (0-8.75 years; 95% CI [1.9–4.2]); *p* = 0.710; regorafenib: 1.8 years (0-5.9 years; 95% CI [1.1–2.6]) vs. 1.9 years (0-3.6 years; 95% CI [1.2–2.5]); *p* = 0.576, respectively, Fig. 2B, D, F, G)

### Overall survival by location and time of metastases

**Fig. 3:**
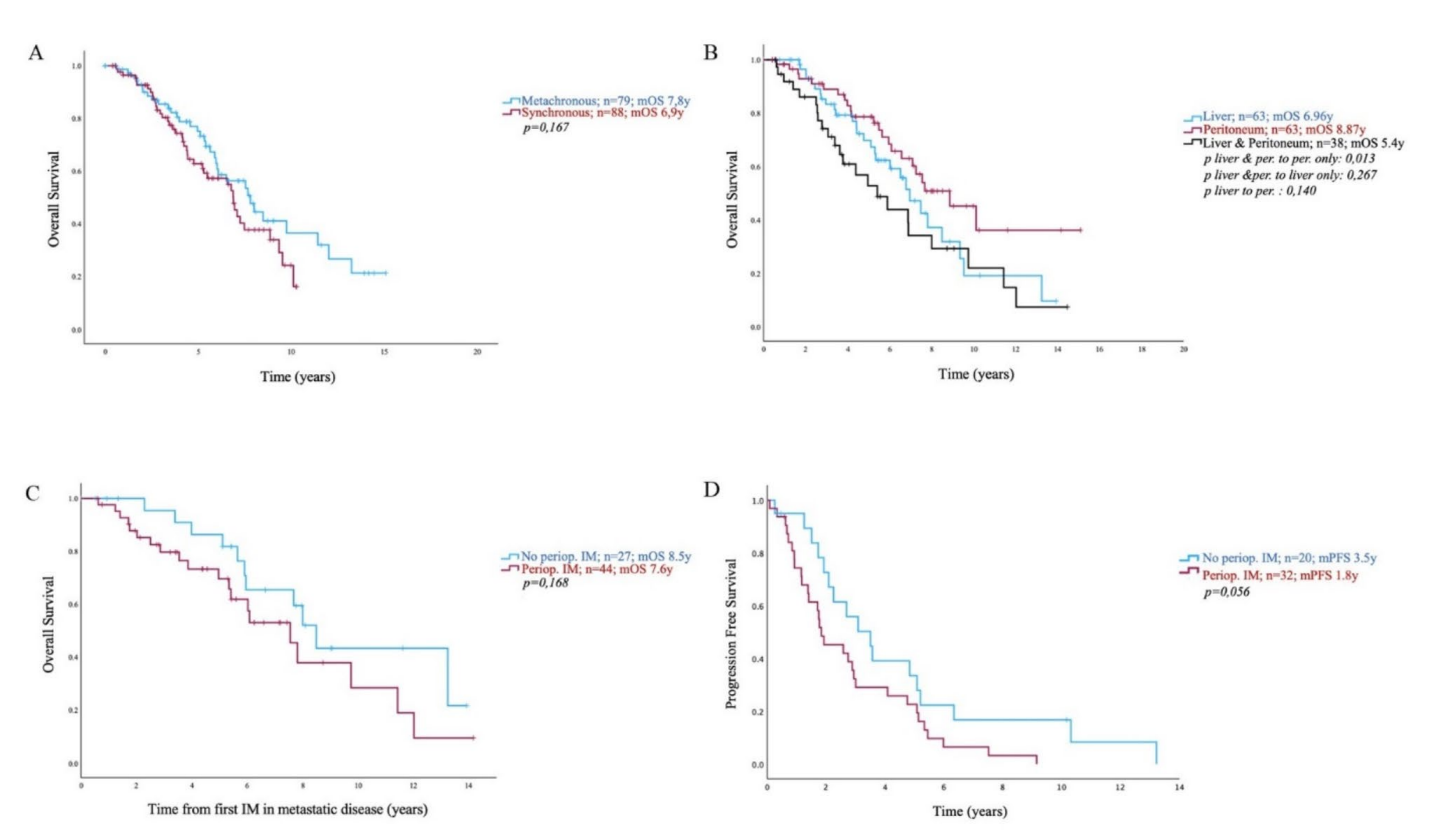
**A** and **B**): Overall survival curve calculated from first treatment with imatinib until last FU or death, based on time to metastases. (3**A**). Overall survival curve calculated from the first treatment with imatinib until last follow-up (FU) or death, based on the location of metastases (3**B**). **C** and **D**): Overall survival curve calculated from the first treatment with imatinib until last FU or death, based on prior perioperative imatinib treatment (3**C**). Time to progression curve calculated from the date of first imatinib for metastatic disease until progression, based on prior perioperative imatinib treatment (3**D**)

Median overall survival in patients with metastases confined to the peritoneum was longer (8.87 years, range 0-15.1 years; 95% CI [6.5–11.2]) compared to those with both hepatic and peritoneal metastases (5.4 years, range 0-14.5 years; 95% CI [3.3–7.51]; *p* = 0.013). No significant difference was found between patients with hepatic (6.96 years, range 0-13.9 years; 95% CI [5.7–8.2]) or peritoneal metastases alone (*p* = 0.140), or hepatic and peritoneal metastases (*p* = 0.267) (Fig. 3B). We further compared overall survival in patients with synchronous versus metachronous metastatic disease, starting from the first palliative treatment with imatinib. A similar outcome was observed for both groups (mOS synchronous metastases: 6.9 years, range 0-10.3 years; 95% CI [5.4–8.4]; mOS metachronous metastases: 7.8 years, range 0-15.1 years; 95% CI [5.99–9.6]; *p* = 0.167) (Fig. 3A).

### Outcome after prior imatinib therapy

Additionally, we compared the progression-free survival of patients following imatinib treatment for metastatic disease against the background of prior perioperative imatinib in localized disease. The median interval between the end of perioperative imatinib and the initiation of treatment for metastatic disease was 13.3 months. The progression-free survival in patients with prior imatinib for localized disease was 1.8 years (range 0-9.1 years; 95% CI [0.8–2.8]) compared to 3.5 years of patients who were therapy-naïve (range 0-13.2 years; 95% CI [1.8–5.2]) but this difference did not reach statistical significance (*p* = 0.056) (Fig. 3D). There was no significant difference in overall survival between the two groups (8.5 years in patients with no perioperative imatinib treatment, range 0-13.9 years; 95% CI [7.2–9.8]; versus 7.6 years in patients with perioperative imatinib treatment, range 0-14.2 years; 95% CI [5.6–9.5]; *p* = 0,168) (Fig. 3C). The length of the interval between the end of perioperative imatinib and the beginning of palliative imatinib did not have an impact on prognosis in this patient cohort (data not shown).

## Discussion

Survival remains one of the most important aspects when consulting patients with metastatic GIST. While published survival data from clinical registration trials and international retrospective analyses are highly relevant, they do not fully represent the ‘real world’ patient conditions or country-specific aspects. Our study provides one of the largest real world up-to-date data sets from a German tertiary care center that has specialized in GIST care for more than 20 years.

**Fig. 4:**
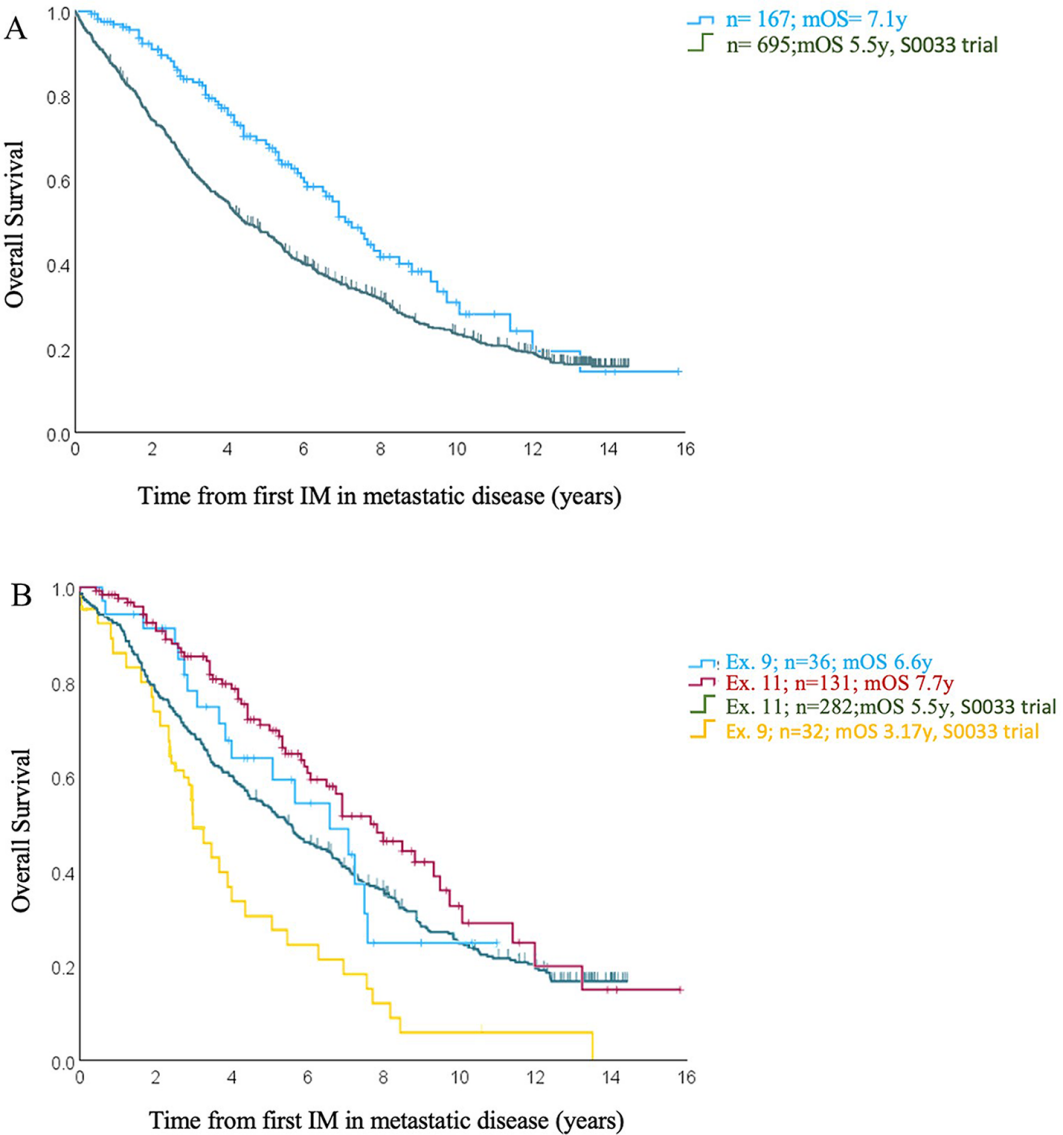
**A** and **B**: Survival analyses calculated from first treatment with imatinib for metastatic GIST to last follow-up (FU) or death within our cohort compared with long-term results of imatinib in advanced GIST, analysis of the phase 3 SWOG Intergroup Trial S0033, excluding wild-type mutations for better comparability (Heinrich et al. 2017)

Patients in our cohort showed longer overall survival compared to historical studies for 1st and 2nd -line treatments. The median OS in our study was 7.1 years (Fig. 2A), compared to 4.1 years reported in the Meta-GIST analysis from 2010, which combined data from two randomized phase III trials comparing two doses of imatinib (MetaGIST 2010). In addition, we compared our data to the analysis by the SWOG Intergroup (Heinrich et al. 2017), which reported a 15-year follow-up study of the S0033 phase III trial. This trial, which completed accrual before 2002, involved treatment-naive patients with metastatic or advanced, surgically unresectable GIST. Comparing the total patient cohort, we observed an overall survival of 7.1 years versus 5.5 years (Fig. 4A), indicating a significant improvement in overall survival in our cohort. In contrast to our cohort, this study also included KIT/PDGFRA wild-type GIST (not otherwise specified), but the overall survival was also improved when focusing on exon 11 and exon 9 genotypes (ex. 11 mOS 7.7 years vs. 5.5 years; ex. 9 mOS 6.6 vs. 3.17 years, respectively, Fig. 4B).

In our cohort, patients starting on sunitinib as second-line treatment showed a mOS of 2.9 years (Fig. 2C), compared to the registration trial in 2012 (1.4 years) (Demetri 2012). The overall survival in the 3rd-line GRID trial was 17.4 months versus 23 months (Fig. 2E) in this cohort (Demetri et al. 2016). However, a phase II trial assessing overall survival in patients treated with regorafenib is aligned with the real-world data that we observed (25 months), likely due to continuous treatment in experienced centers (Ben-Ami et al. 2016).

Registration studies for imatinib date back more than 20 years, to a time when patients were more likely to start treatment with more advanced disease due to the limited availability of imatinib, which was then the only approved treatment. For localized high-risk tumors, follow-up has become more standardized in many centers over the past decade, leading to earlier detection of relapses and subsequently a lower tumor burden in patients with metachronous disease. In addition, registration studies for second- and third-line treatments have recruited patients from a wide range of health care systems, many of which do not provide immediate reimbursement for approved drugs or universal coverage. In contrast, our cohort comes from a health care system that offers universal coverage and early access to approved drugs.

Plenty of evidence suggests, that patient care by specialized teams improves the outcomes for patients with sarcomas, including GIST (Blay 2017; Blay 2019). Several factors likely contribute to improved outcomes: diligent and standardized radiological follow-up, radiological expertise (to identify e.g. pseudo-progression), side effect management by experienced staff to maximize the use of treatment lines, multimodal treatment approaches, and access to clinical trials (Casali et al. 2022).

In our data set, the median time from the first time of diagnosis to first visit at our center was 27 months (0-290months, Table 1). As in most countries, primary treatment is predominantly conducted in local hospitals (87.9%, Table 1). Notably, at time of diagnosis of metastatic disease the involvement of sarcoma centers increased to 36.8% (Table 1).

In Germany, sarcoma centers are designated based on a well-defined quality control measures as well as minimum patient numbers. However, criteria that define a “GIST center of excellence” have not been established yet, but obviously the number of patients and access to clinical trials should represent the main criteria.

Notably, 6 patients (7.8%, Table 1) in our cohort died of non-GIST related causes, primarily due to cardio-vascular events. These deaths may be attributed to lifestyle factors or age, but side effects from long-term TKI treatment should also be considered.

In our cohort, the median time from diagnosis of GIST to the diagnosis of metastatic disease was five months (Table 1). 51.1% of patients had their diagnosis of metastases within the first six months after their GIST diagnosis (defined as synchronous, Table 1). While early metastases could suggest more aggressive disease and potentially higher tumor burden, we did not observe a difference in overall survival (Fig. 3A). This lack of difference may be explained by the biological relevance and oncogenic dependency of KIT. Imatinib and other TKIs appear to be equally effective regardless of the timing of diagnosis.

Although GIST primarily arise in the stomach, our cohort highlights the importance of the primary tumor’s location as a risk factor for metastatic disease, given the predominance of small intestine-primary tumors in this cohort (Table 1) (Miettinen 2006; Fernández 2021). We also examined the importance of metastatic sites at the time of diagnosis of metastases. Patients with both hepatic and peritoneal metastases showed a mOS (as calculated from first imatinib for metastatic disease) of 5.4 years compared to 8.87 years in patients with peritoneal metastases only (*p* = 0.013, Fig. 3B). The prognosis of patients with metastatic GIST may depend on various factors, including tumor burden. In addition, surgical approaches may have a profound impact on survival in patients in whom complete macroscopic resection can be achieved (Bauer 2014). In our cohort, 39.6% of patients who underwent metastasectomy had metastases limited to the peritoneum. However, the number of patients receiving local treatment and the depth of data regarding surgical procedures preclude further conclusions on the impact of surgery in the context of organ involvement.

Patients frequently ask about the relevance of perioperative imatinib for promoting resistance when treated for metastatic disease. In this cohort, progression-free survival in patients who received prior imatinib treatment (1.8 years, range 0-9.1 years) was close, barely not significantly different, from that of the therapy-naïve patients (3.5 years, range 0- 13.2 years; *p* = 0.056, Fig. 3D). In addition, there was no significant difference in overall survival (*p* = 0,168, Fig. 3C).

The French BFR14 study recently updated its long-term survival analysis for patients who discontinued treatment with imatinib for metastatic disease. According to this study, overall survival was significantly shorter in patients who discontinued treatment compared to those who continued treatment (Blay et al. 2024). Even if there is no significant difference in overall survival within our cohort, there are indications of relevance in progression-free survival. Possible confounding factors, such as the risk of relapse, must be considered in this context. In our cohort, 58.4% of patients who received imatinib perioperatively had a relapse risk of at least 60% for the primary disease, whereas only 29.6% of patients who did not receive imatinib had a relapse risk above 60% (Table 1).

## Conclusion

In conclusion, this study provides a novel, real-world reference for survival in patients with metastatic GIST. The reason for the observed survival exceeding historical data can only be speculated about. Likely factors include a lower tumor burden, broader access to salvage agents, as well as the availability of experienced treatment teams and patient awareness. The recent approval of the first drug specifically developed for GIST (Blay et al. 2020) along with other drugs and drug combinations (De Sutter et al. 2023; Blum et al. 2023; Wagner et al. 2021) with a broader inhibitory spectrum, are expected to further improve the outcomes of patients with metastatic GIST.

## Data Availability

No datasets were generated or analysed during the current study.
